# Postoperative outcomes after inguinal hernia repair in octogenarians in Germany: an exploratory analysis of a retrospective single-centre cohort

**DOI:** 10.1007/s00423-026-04244-7

**Published:** 2026-09-11

**Authors:** Hanan El youzouri, Yvonne Beaugé, Kim L. Zerr, Michel Kostantin, Safoora Cheema, Armin Wiegering, Teresa Schreckenbach

**Affiliations:** 1 Department of General- and Visceral Surgery, Goethe University Frankfurt am Main, Theodor-Stern-Kai 7, 60528 Frankfurt am Main, Germany; 2 Medical Faculty, Institute for Medical Education and Clinical Simulation, Goethe University Frankfurt, Theodor-Stern Kai 7, 60590 Frankfurt/Main, Germany

**Keywords:** Inguinal hernia repair, Octogenarians, Postoperative complications, Frailty

## Abstract

**Background:**

As the population ages, the number of elderly patients undergoing inguinal hernia repair is increasing. Advanced age is associated with higher rates of comorbidity and frailty, which may potentially affect postoperative outcomes. This study evaluated postoperative morbidity in different age groups with a focus on octogenarians.

**Methods:**

This exploratory retrospective single-centre study included 388 patients undergoing elective inguinal hernia repair between 2014 and 2020. The patients were stratified into three groups according to age < 65 years (*n =* 240), 65–79 years (*n =* 117), and ≥ 80 years (*n =* 31). Open (Lichtenstein) and transabdominal preperitoneal laparoscopic techniques were analysed. The primary outcome was postoperative morbidity according to the Clavien–Dindo classification. Logistic regression analyses were performed to identify risk factors.

**Results:**

Octogenarians more frequently underwent open repair, whereas younger patients more often received laparoscopic treatment (*p <* 0.001). No significant differences were observed between age groups in regard to intraoperative or overall postoperative complications, including complication severity. In the multivariable analysis, age was not associated with postoperative complications (*p =* 0.974). Laparoscopic repair was associated with lower odds of postoperative complications (odds ratio (OR) 0.544; *p =* 0.031), while longer operative time was associated with increased risk of complications (OR 1.009; *p =* 0.022).

**Conclusions:**

Octogenarians did not show increased postoperative morbidity. Surgical decision-making should not rely on chronological age alone.

**Trial registration:**

Not applicable.

## Background

 Life expectancy in Germany has had a long-term upward trend for both women and men, despite a temporary decline during the COVID-19 pandemic [1, 2]. Thus, the absolute number and proportion of octogenarians in the population is increasing, and this demographic shift is accompanied by a growing number of elderly patients presenting for elective surgical procedures [3]. Accordingly, an increasing number of male patients aged 80 years and older are undergoing elective inguinal hernia repair, which poses specific clinical and perioperative challenges [4].

With advancing age, there is a greater burden of chronic comorbid conditions, such as cardiovascular disease, diabetes mellitus, and neurocognitive disorders increases, which contribute to a higher prevalence of frailty in older surgical patients [5]. Cardiovascular disease and the subclinical cardiovascular burden are highly prevalent in adults aged 80 years and older. The prevalence of diabetes also increases with age and is linked to accelerated cardiovascular and functional deterioration [6–8]. Therefore, patients aged 80 years and older may be more likely to exhibit features associated with frailty, which may potentially increase perioperative risk and complexity [5]. On the other hand, a subset of the “oldest old” demonstrates relatively preserved functional status and resilience due to selective survival and adaptive physiologic reserve, which has been described in aging research as an aspect of the marked heterogeneity in health and functional reserve among this group [9, 10].

Inguinal hernias are one of the most common conditions encountered in general surgery, and the lifetime prevalence markedly higher for men than women [11]. Population-based data suggest that approximately 27% of men will develop an inguinal hernia during their lifetime, whereas the corresponding lifetime risk for women is around 3%, which highlights the substantial sex disparity in the disease burden [11]. In symptomatic patients, elective surgical repair is commonly performed to relieve discomfort and improve quality of life. In asymptomatic or minimally symptomatic men, watchful waiting is an accepted option because the risk of hernia-related emergencies is low, although many patients ultimately proceed with surgery because of increasing symptoms [12, 13].

With demographic aging and the increasing prevalence of inguinal hernias in older men, the number of octogenarians undergoing inguinal hernia repair is expected to rise. This development underscores the need for better understanding of the perioperative outcomes and risk profiles in this expanding patient population. The aim of this retrospective study was to compare postoperative outcomes after inguinal hernia repair among patients aged < 65 years, 65–79 years, and ≥ 80 years. Using real-world data, the particular focus was on exploring whether advanced chronological age was associated with increased postoperative morbidity. To provide a realistic representation of daily clinical practice, the analysis included both Lichtenstein and transabdominal preperitoneal (TAPP) laparoscopic repair techniques, as well as patients with unilateral, bilateral, and recurrent inguinal hernias.

## Methods

### Patient demographics and clinical data

This retrospective cohort study was conducted at Frankfurt University Hospital. Between January 1, 2014 and December 31, 2020, 401 patients underwent open or laparoscopic inguinal hernia repair. Data were collected retrospectively and analysed in accordance with the STROBE guidelines [14]. Patients with primary or recurrent, unilateral or bilateral inguinal hernia were included, while those who underwent emergency procedures were excluded (*n* = 13). The final study cohort comprised 388 patients. Ethical approval for the study was granted by the Ethics Committee of the Faculty of Medicine at Goethe University Frankfurt (approval number 2022/788).

**Fig. 1:**
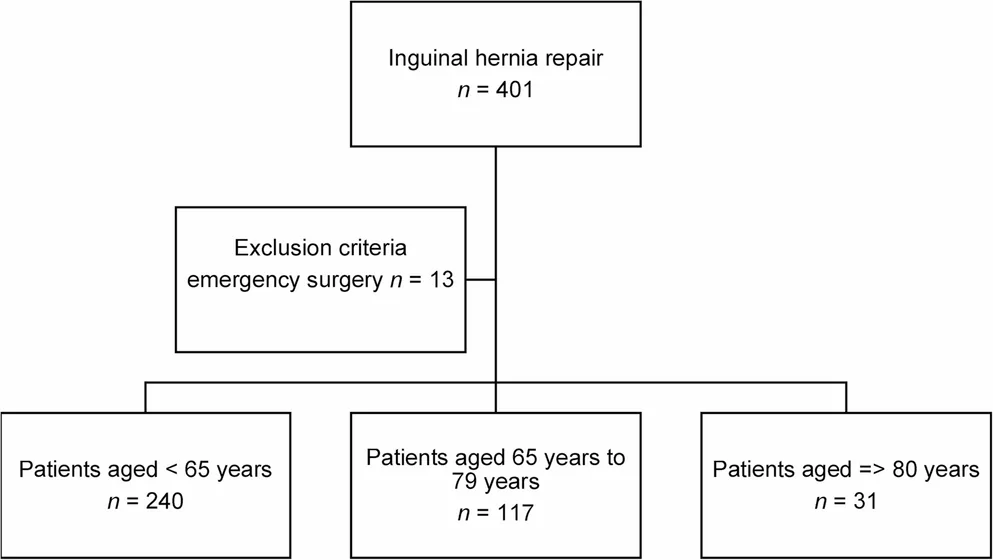
Flow chart

The variables recorded included demographic and clinical characteristics such as age, sex, body mass index (BMI, kg/m²), pre-existing comorbidities (coronary artery disease, chronic obstructive pulmonary disease, liver cirrhosis, renal insufficiency, diabetes mellitus, and dementia), American Society of Anesthesiologists (ASA) classification, relevant risk factors (including smoking, viral hepatitis, HIV infection, and alcohol use), and history of prostate cancer. ASA classification was used as a surrogate parameter for perioperative health status. Frailty-specific or functional status variables were not routinely assessed in this retrospective cohort. Patients were stratified into three predefined age groups to compare younger, older, and octogenarian patients (< 65 years, 65–79 years, and ≥ 80 years). The respective group sizes were 240, 117, and 31 patients. Further details are presented in Fig. 1.

A total of 38 surgeons performed the 388 inguinal hernia procedures included in the study. Case volume was unevenly distributed among surgeons: one surgeon performed 99 procedures (25.5%), while five additional surgeons each performed more than 20 procedures, collectively accounting for another 148 procedures. Thus, six surgeons performed 247 of 388 procedures (63.7%). The remaining procedures were performed by board-certified surgeons and surgical residents under the supervision of experienced surgeons.

## Complications

Complications were divided into intraoperative complications and postoperative complications. Intraoperative complications included bowel injury and injury of the spermatic cord or testicular vessels (in male patients). Postoperative complications included surgical site infections (SSIs), hematoma, seroma, numbness, chronic pain, and reoperation. Complications were graded according to the Clavien–Dindo classification (CDC) [15].

## Study endpoints and statistical analyses

The primary outcome was the occurrence of any postoperative complication, which was defined according to CDC grade ≥ I. Particular focus was placed on postoperative morbidity in octogenarian patients in comparison with younger age groups. For multivariable analysis, age was also analysed as a continuous variable. Secondary outcomes included operation duration, length of hospital stay, and hernia recurrence rate. Hernia recurrence was defined as a recurrent inguinal hernia documented in the medical records during the follow-up period.

Statistical analyses were performed using IBM SPSS Statistics for Windows (version 29; IBM, Chicago, IL, USA). Categorical variables were summarized as absolute numbers and percentages, while continuous variables were reported as means with the standard deviations (SDs) or medians with interquartile ranges (IQRs) as appropriate. Group comparisons between octogenarians and younger patients were conducted using the chi-squared (*χ*²) test or Fisher’s exact test for categorical variables and one-way analysis of variance (ANOVA) or the Mann–Whitney U test for continuous variables, depending on the data distribution. The strength of associations between categorical variables was assessed using Cramer’s *V* and interpreted according to Cohen’s conventions (small effect: 0.1, medium effect: 0.3, large effect: 0.5) [16].

Univariable logistic regression analysis was performed to identify factors associated with any postoperative complications (CDC). The following variables were included a priori based on their potential clinical relevance: age, gender, ASA score, comorbidities, BMI, smoking status, history of prostatectomy, type of surgery, and duration of surgery. The results are reported as odds ratios (ORs) with corresponding 95% confidence intervals (CIs). A *p*-value < 0.05 was considered statistically significant.

A multivariable logistic regression analysis was then performed to evaluate independent predictors of any postoperative complications. All variables included in the univariable analysis were also entered into the multivariable model to adjust for potential confounding effects. Prior to model construction, multicollinearity between variables was assessed, and no relevant collinearity was detected.

## Results

### Patient characteristics

Between January 2014 and December 2020, there were 401 inguinal hernia repairs performed in our department. After exclusion of 13 cases, 388 patients were included in the analyses. 229 of the surgeries involved laparoscopy (TAPP), and 159 involved open repairs (Lichtenstein). The mean age of the study population was 58.5 ± 16.7 years. In the whole cohort, 31 patients (7.99%) were 80 years of age or older. Table 1 shows the cohort details.

**Table 1:** Patient characteristics. Categorical variables were compared using the chi-squared test or Fisher’s exact test as appropriate (indicated by *). Pairwise group comparisons were adjusted using Bonferroni correction. Continuous variables were analysed using one-way analysis of variance (ANOVA) with Bonferroni post-hoc correction

	All *N* = 388	Patients aged < 65 years *N* = 240	Patients aged 65 years to 79 years *N* = 117	Patients aged ≥ 80 years *N* = 31	Pearson Chi-Squared (df)	Cramer’s V	*P*
Gender Male Female	367 (94.6) 21 (5.4)	225 (93.8) 15 (6.3)	115 (98.3) 2 (1.7)	27 (87.1) 4 (12.9)	6.86 (2)	0.133	0.032
Comorbidities Cardiovascular disease COPD Liver cirrhosis CKD on dialysis CKD without dialysis Diabetes mellitus Dementia	179 (46.1) 18 (4.6) 19 (4.9) 4 (1.0) 28 (7.2) 40 (10.3) 6 (1.5)	72 (30.0) 7 (2.9) 10 (4.2) 3 (1.3) 11 (4.6) 16 (6.7) 1 (0.4)	80 (68.4) 9 (7.7) 8 (6.8) 1 (0.9) 13 (11.1) 17 (14.5) 2 (1.7)	27 (87.1) 2 (6.5) 1 (3.2) 0 4 (12.9) 7 (22.6) 3 (9.7)	69.4 (2) 4.31 (2) 1.41 (2) 0.47 (2) 6.63 (2) 10.75 (2) 15.49 (2)	0.423 0.105 0.060 0.035 0.131 0.166 0.200	**< 0.001** 0.116* 0.495* 0.790* 0.036* 0.005* < 0.001*
Prostatic cancer in the last 5 Years Yes Prostatectomy Yes	17 (4.4) 15 (3.9)	0 0	16 (94.1) 14 (93.3)	1 (3.2) 1 (3.2)	35.22 (2) 30.34 (2)	0.301 0.280	< 0.001* < 0.001*
Risk factors Smoking Alcoholism HIV Viral hepatitis	98 (25.3) 35 (9.0) 9 (2.3) 13 (3.8)	81 (33.8) 17 (7.1) 7 (2.9) 9 (3.1)	16 (13.7) 15 (12.8) 2 (1.7) 2 (1.7)	1 (3.2) 3 (9.7) 0 2 (6.5)	25.45 (2) 3.17 (2) 1.31 (2) 2.01 (2)	0.256 0.090 0.058 0.072	**< 0.001** 0.205* 0.520* 0.366*
ASA score (*n =* 384) ASA 1–2 ASA ≥ 3	268 (69.8) 116 (30.2)	196 (82.7) 41 (17.3)	61 (52.6) 55 (47.4)	11 (35.5) 20 (64.5)	52.33	0.369	**< 0.001**
	**All** ***n =*** **388**	**Patients aged < 65 years** *N* = 240	**Patients aged 65 years to 79 years** *N* = 117	**Patients aged = > 80 years** *N* = 31	***F***		***P***
Age in years (mean±SD)	58.5(±16.7)	48.2 (±12.0)	72.0 (±4.3)	85.1 (±3.9)	366.04		**< 0.001**
BMI (kg/m^2^) (mean±SD)	25.2 (±4.2)	25.5 (±4.5)	25.0 (±3.8)	24.3 (±3.4)	1.35		0.260
	COPD: chronic obstructive pulmonary disease, CKD: Chronic kidney disease, BMI: body mass index, SD: standard deviation, HIV: human immunodeficiency virus, ASA: American Society of Anesthesiologists						

### Surgical details

In the entire cohort, 12.7% of patients underwent inguinal hernia repair due to recurrent hernia, but none were in the octogenarian group. Minimally invasive hernia repair was performed for 70.8% of the younger patients, 41.9% of the middle-aged group, and 32.2% of the octogenarian group, and the differences were statistically significant (*p* < 0.001). The operation time was significantly longer for TAPP than the Lichtenstein procedure (79.6 ± 31.3 vs. 66.6 ± 31.1 min, Mann–Whitney *U* test, *p* < 0.001). There were no statistically significant differences in overall intraoperative or postoperative complications between groups. Although no significant overall differences in intraoperative or postoperative complications were observed between the age groups, pairwise comparisons demonstrated significantly higher rates of SSIs and complications with CDC grade I in patients aged ≥ 80 years compared to those aged < 65 years (*p* < 0.05). More details are shown in Table 2.

**Table 2:** Surgical details. Categorical variables were analysed using the chi-squared test or Fisher’s exact test as appropriate (*). Pairwise comparisons were adjusted using the Bonferroni correction. Continuous variables were analysed using one-way analysis of variance (ANOVA) with Bonferroni post-hoc correction. Superscript letters (a, b) indicate statistically significant differences between groups (*p* < 0.05) in post-hoc pairwise comparisons; groups sharing the same letter are not significantly different. The symbol † indicates overall non-significant differences between groups with significant differences detected in pairwise comparisons (*p <* 0.05)

	Total (%) *n =* 388	Patients aged < 65 years *N* = 240		Patients aged 65 years to 79 years *N* = 117		Patients aged ≥ 80 years *N* = 31		Pearson Chi-Squared (df)	Cramer’s V		*P*
Recurrent herniayes	49 (12.7)	30 (12.6)		19 (16.2)		0		5.85 (2)	0.123		0.054*
Bilateral hernia yes	34 (8.8)	22 (9.2%)		10 (8.6)		2 (6.5)		0.134 (2)	0.018		0.935
Surgical technique Lichtenstein TAPP	159 (41.0) 229 (59.0)	70 (29.2) 170 (70.8)		68 (58.1) 49 (41.9)		21 (67.7) 10 (32.2)		37.239 (2)	0.310		**< 0.001**
Anaesthesia (for Lichtenstein procedure *n* = 159, missing *n* = 2) General Regional/Local	145 (92.4) 12 (7.6)	66 (34.3) 4 (5.7)		62 (93.9) 4 (6.1)		17 (81.0) 4 (19.0)		4.472 (2)	0.169		0.107
Intraoperative complications yes	4 (1.0)	3 (1.3)		1 (0.9)		0		0.47 (2)	0.035		0.790*
Intraoperative complications Bowel injury Injury of spermatic cord	2 (0.5) 2 (0.5)	1 (0.4) 2 (0.8)		1 (0.9) 0		0 0		0.47 (2) 1.24 (2)	0.035 0.057		0.791* 0.538*
Postoperative complications Surgical site infection Hematoma Seroma	8 (2.1) 16 (4.1) 5 (1.3)	2 (0.8)^a^ 8 (3.3) 1 (0.4)		4 (3.4)^a, b^ 6 (5.1) 3 (2.6)		2 (6.5)^b^ 2 (6.5) 1 (3.2)		5.819 (2) 1.103 (2) 3.845 (2)	0.122 0.053 0.100		0.055^†^ 0.576 0.146
Clavien–Dindo Classification CDC I CDC II CDC III CDC IV CDC V	19 (4.9) 12 (3.1) 6 (1.5) 1 (0.3) 0	8 (3.3)^a^ 4 (1.7) 5 (2.1) 1 (0.4) 0		7 (6.0)^a, b^ 7 (6.9) 1 (0.9) 0 0		4 (12.9)^b^ 1 (3.2) 0 0 0		12.58 (8)	0.127		0.127^†^
Other Complications Persistent pain > 3 months Numbness > 3 months	13 (3.4) 8 (2.1)	10 (4.2) 7 (2.9)		2 (1.7) 1 (0.9)		1 (3.2) 0		1.47 (2) 2.37 (2)	0.062 0.078		0.480* 0.306*
Recurrent hernia after last operation yes	8 (2.1)	6 (2.5)		2 (1.7)		0		0.95 (2)	0.050		0.621*
	**Total (%)** ***n =*** **388**		**Patients aged < 65 years** *N* = 240		**Patients aged 65 years to 79 years** *N* = 117		**Patients aged = > 80 years** *N* = 31		**F-value**	**P-value**	
Operation time in minutes (mean±SD)	74.2 (±31.8)		77.2 (±32.7)		71.2 (±31.5)		62.7 (±21.3)		3.63	**0.028**	
Hospital stay in days (mean±SD)	2.3 (±2.0)		2.2 (±2.3)		2.2 (±1.2)		3.4 (±2.9)		4.37	**0.013**	
Follow up in months (mean±SD)	27.0 (±30.5)		25.0 (±29.9)		33.3 (±31.8)		20.8 (±27.4)		3.26	**0.040**	

## Logistic regression analysis

A logistic regression analysis was performed to identify factors associated with postoperative complications classified according to the CDC criteria. In the univariable analysis, a history of prostatectomy was identified as a significant risk factor for postoperative complications (OR 3.594; 95% CI 1.27–10.19; *p* = 0.016). In contrast, the use of TAPP was associated with lower odds of complications (OR 0.587; 95% CI 0.37–0.93; *p =* 0.024).

In the multivariable logistic regression analysis, TAPP remained independently associated with lower odds of postoperative complications (OR 0.544; 95% CI 0.31–0.95; *p =* 0.031). Additionally, longer duration of surgery was identified as a risk factor for postoperative complications (OR 1.009; 95% CI 1.00–1.02; *p =* 0.022). Age as a continuous variable did not show a statistically significant association with postoperative complications in the multivariable analysis (*p =* 0.974). Detailed results are presented in Table 3.

**Table 3:** Univariable and multivariable logistic regression analysis of risk factors for any postoperative complications

	Any Complication According to CDC							
	Univariate Logistic Regression				Multivariate Logistic Regression			
	OR	95% CI	*B* coefficient *(df)*	*P*	OR	95% CI	*B *coefficient *(df)*	*P*
Age	1.008	0.99–1.02	*0.008 (1)*	*0.252*	*1.000*	*0.982–1.02*	0.000	*0.974*
Gender Male	0.836	0.32–2.22	-0.179 (1)	0.720	0.829	0.28–2.47	-0.188	0.736
ASA Score ASA$\:\ge\:\:$3	1.407	0.86–2.30	0.341 (1)	0.173	1.263	0.67–2.38	0.234	0.470
Comorbidities Cardiovascular disease Diabetes mellitus COPD	1.231 0.599 0.579	0.78–1.95 0.26–1.40 0.16–2.04	0.208 (1) -0.521 (1) -0.547 (1)	0.375 0.237 0.396	1.000 0.510 0.407	0.54–1.87 0.21–1.27 0.10–1.60	0.000 -0.673 -0.898	1.000 0.148 0.198
BMI (kg/m^2^)	1.005	0.95–1.06	0.005 (1)	0.852	0.997	0.94–1.06	-0.003	0.929
Smoker Yes	0.697	0.40–1.22	-0.361 (1)	0.203	0.763	0.42–1.38	-0.271	0.371
Prostatectomy in anamnesis Yes	3.594	1.27–10.19	1.279 (1)	**0.016**	2.925	0.95–8.99	1.073	0.061
Type of surgery TAPP	0.587	0.37–0.93	-0.533 (1)	**0.024**	0.544	0.31–0.95	-0.609	**0.031**
Duration of surgery (min)	1.006	0.99–1.01	0.006	0.104	1.009	1.00-1.02	0.009	**0.022**

## Discussion

This retrospective single-centre analysis evaluated the postoperative outcomes following surgical treatment of inguinal hernia in younger and middle-aged patients in comparison with octogenarians. The findings suggest that elective inguinal hernia repair was not associated with higher postoperative morbidity in the selected cohort of patients aged 80 years and older in comparison younger patients. Furthermore, a significantly higher proportion of octogenarians underwent open herniotomy, which reflects real-world clinical practice.

As life expectancy rises and the quality of life for older adults improves, the proportion of octogenarians who require elective non-oncological surgical treatment is also increasing. Huerta et al. analysed a cohort of 109 veterans, who were mostly aged 80 years or older [17]. They showed that short-term mortality was not increased in patients undergoing inguinal hernia repair, although they also demonstrated that general anaesthesia should be avoided [17]. However, no comparisons were made with younger patients. Ding et al. observed no significant differences in postoperative complications between octogenarians and younger patients undergoing open or laparoscopic groin hernia repair. Nevertheless, they did observe a significantly higher rate of systemic complications in octogenarians undergoing laparoscopic repair compared to open repair [18].

The present study also did not indicate differences between age groups, but laparoscopic repair was associated with lower odds of postoperative complications in the overall cohort. Although laparoscopic repair appeared to be a protective factor against postoperative complications in this cohort, this observation should be interpreted cautiously in octogenarians as only 10 patients aged ≥ 80 years underwent TAPP repair. Thus, it was not feasible to perform a robust subgroup analysis comparing laparoscopic and open repair in octogenarians, and no definitive recommendation can be made in regard to the optimal surgical approach for this age group based on the present data.

There are two factors that could negatively influence postoperative outcomes for laparoscopic repair of hernia in octogenarians: the need for general anaesthesia and the requirement for pneumoperitoneum. Mizuno et al. examined 1935 octogenarians and analysed the best surgical and anaesthetic approach for hernia repair [19]. They found that the laparoscopic approach is safe and feasible for elderly patients, and local aesthesia was associated with favourable perioperative outcomes in open surgery [19]. Other studies have shown that in laparoscopic colorectal surgery for octogenarians, shorter operative time and minimal blood loss are key factors for reducing postoperative complications [20, 21]. However, these findings are only indirectly transferable to inguinal hernia repair due to differences in surgical complexity and perioperative risk profiles.

For laparoscopic and open hernia repair, blood loss is generally minimal and unlikely to be a major risk factor. The operation time can differ significantly between Lichtenstein procedure and TAPP, as demonstrated in the present study, and prolonged operation time may indicate intraoperative difficulties and contribute to postoperative complications. In the present cohort, octogenarians had shorter operation times overall despite TAPP procedures taking longer than Lichtenstein repair, which suggests potential confounding by the selection of the surgical approach and patients. For example, longer operation time or larger incisions may lead to greater risk of SSIs [22]. Christou at al. examined 21,976 patients and analysed potential risk factors for surgical site infections after groin hernia surgery and did not observe a significant influence of mesh type or surgical technique on SSI rates. As the overall SSI rate was 0.26%, the authors stated that the results should be interpreted with caution [23].

The improvement in quality of life after inguinal hernia repair must justify the potential risks in elderly patients. Although quality of life and patient-reported outcomes were not assessed in the present study, these aspects are highly relevant for clinical decision-making for geriatric patients. Collins at al. analysed quality of life as an outcome after ventral hernia repair and found comparable improvements even in very elderly patients [24]. Schoel et al. asked older patients who underwent ventral, incisional, or inguinal hernia repair whether they regretted their decision to undergo surgery [25]. 795 patients were included, and although 16% of these patients were severely frail, frailty was not associated with regret about the surgery [25].

However, findings from ventral and incisional hernia repair may only be partially transferable to inguinal hernia surgery because of differences in operative complexity, postoperative pain, and recurrence risk. This raises the question of whether it is justified to apply a “watch-and-wait” strategy, which is sometimes employed for symptomatic or minimally symptomatic inguinal hernia. Van den Dop demonstrated that among men over 50 years of age, 64.2% of patients in the watch-and-wait group eventually required surgery over a 12-year follow-up period [26]. This should be given particular consideration for older patients as outcomes following emergency surgery, such as for incarcerated hernias, can be significantly worse. For example, Bal et al. demonstrated that elderly patients who underwent emergency treatment for an inguinal hernia experienced significant postoperative functional decline [27].

It should be noted that emergency procedures were excluded from the present study, so they were not represented in the analysed cohort. Other studies show that comorbidities such as diabetes, cardiovascular diseases, and chronic obstructive pulmonary disease (COPD), should be taken more into account when deciding whether or not to perform surgery rather than age alone [18]. In the present study, no significant independent effects of these comorbidities were observed, which may have been due to the limited sample size and statistical power. In addition to comorbidities, frailty has emerged as an important predictor of postoperative outcomes in elderly surgical patients and may provide a more accurate risk stratification than chronological age alone [28]. Therefore, future studies should incorporate standardized frailty and functional status assessments whenever possible. From a clinical perspective, the relevance of the present findings lies less in the feasibility of inguinal hernia surgery itself and more in risk assessment and surgical decision-making in very elderly patients. Our findings support the concept that chronological age alone may be insufficient for this purpose, although the limited sample size precludes definitive conclusions.

Accordingly, surgical treatment decisions for elderly patients should be based on shared decision-making that incorporates frailty, functional status, comorbidities, patient goals, expected quality of life, and the potential risk of emergency surgery. Similar observations have also been reported for other abdominal surgical procedures, although these findings are not directly transferable to inguinal hernia repair. In colorectal surgery, chronological age alone was not identified as an independent risk factor for adverse postoperative outcomes, whereas failure-to-rescue rates, comorbidity burden, and perioperative risk profiles appeared to be more relevant determinants of outcome [29, 30].

The specific healthcare setting and study period should also be considered when interpreting the present findings. During the study period from 2014 to 2020, reimbursement structures and clinical pathways for inguinal hernia surgery in Germany differed from healthcare systems in which day-case surgery had already been more widely implemented. Since completion of the study period, the German reimbursement framework has undergone substantial changes, including the introduction of Hybrid-Diagnosis related Groups (DRG) reimbursement for selected hernia procedures [31]. Consequently, the present cohort reflects surgical practice before this transition and should not be interpreted as representative of current outpatient hernia care in Germany.

### Limitations

There are several limitations to this study. The study involved a retrospective single-centre analysis with a limited sample size, particularly for the subgroup of octogenarians, which comprised only 31 patients, whereas 117 middle-aged and 240 younger patients were included. This imbalance between groups limits the statistical power, particularly for subgroup analyses for elderly patients, and results in less precise estimates with wider confidence intervals, thereby restricting the generalizability of the findings. Given the limited sample size, particularly in the octogenarian subgroup, the statistical analyses should be regarded as exploratory, and the absence of statistically significant differences should not be interpreted as evidence of equivalence between the age groups. In addition, the involvement of multiple surgeons with different individual case volumes may have introduced surgeon-related heterogeneity that could not be fully accounted for in this retrospective analysis.

No formal frailty assessment was available, and ASA classification and comorbidity burden may not adequately reflect the functional and physiological vulnerability of elderly patients. Frailty has been shown to be an important predictor of postoperative outcomes in elderly patients, so the absence of such data limits the ability to adequately characterize the physiological status of the octogenarian cohort and may have led to residual confounding. In addition, no subgroup analysis was conducted to compare outcomes between laparoscopic TAPP and open Lichtenstein repair in octogenarians in particular. Therefore, it remains unclear whether one surgical approach may be more beneficial than the other in this particularly vulnerable patient population. The observed association between TAPP repair and lower postoperative morbidity in the overall cohort should not be interpreted as a specific recommendation for patients aged ≥ 80 years. Furthermore, referral and patient-selection patterns at a tertiary university hospital may differ from those in other healthcare settings, which may further limit the generalizability of the findings.

Limitations may also arise from the statistical methodology. Although a multivariable analysis was performed to adjust for potential confounders, residual confounding cannot be excluded. Propensity-score matching was not performed as it would have substantially reduced the sample size and further limited the statistical power of the study. Thus, these limitations should be considered when interpreting the results, and further prospective studies with larger cohorts, standardized frailty assessment, and more detailed subgroup analyses are warranted to validate these findings.

## Conclusion

In this retrospective single-centre cohort, elective inguinal hernia repair was not associated with increased postoperative morbidity in the selected group of octogenarians compared with younger patients. Chronological age alone was not identified as an independent risk factor for adverse postoperative outcomes. Laparoscopic TAPP repair was associated with lower postoperative morbidity in the overall cohort, although it was less frequently performed in older patients, reflecting clinical practice during the study period. Given the limited number of octogenarians, these findings should be considered exploratory. Nevertheless, they support an individualized approach to surgical decision-making in elderly patients that considers individual risk factors, comorbidities, and overall physiological status, including frailty and functional status rather than chronolocical age alone.

## Data Availability

The datasets generated and analysed in the current study are not publicly available due to the ethics committee’s restrictions but are available from the corresponding author upon reasonable request.

## Declarations

### AI statement

The original version of the manuscript was professionally edited for language by American Manuscript Editors. The authors used AI-based language tools, including DeepL, Google Translate, and ChatGPT, to assist with translation and language refinement of selected parts of the manuscript during the revision process. These tools were used solely to improve clarity and linguistic quality and were not used for the generation of scientific content, data analysis, or interpretation. The authors take full responsibility for the content of this manuscript.
